# Potential Role of Membrane Contact Sites in the Dysregulation of the Crosstalk Between Mitochondria and Lysosomes in Alzheimer’s Disease

**DOI:** 10.3390/ijms26209858

**Published:** 2025-10-10

**Authors:** Giulia Girolimetti, Matteo Calcagnile, Cecilia Bucci

**Affiliations:** Department of Experimental Medicine (DiMeS), University of Salento, Via Provinciale Lecce-Monteroni 165, 73100 Lecce, Italy; matteo.calcagnile@unisalento.it

**Keywords:** Alzheimer’s disease, mitochondria, lysosomes, membrane contact site

## Abstract

Alzheimer’s disease (AD) is a neurodegenerative disorder characterized by a gradual decline in cognitive abilities and a progressive loss of the neuronal system resulting from neuronal damage and death. The maintenance of neuronal homeostasis is intricately connected to the crosstalk and balance among organelles. Indeed, intracellular organelles are not just isolated compartments in the cell; instead, they are interdependent structures that can communicate through membrane contact sites (MCSs), forming physical connection points represented by proteinaceous tethers. Mitochondria and lysosomes have fundamental physiological functions within neurons, and accumulating evidence highlights their dysfunctions as AD features, strongly associated with the neurodegenerative process underlying the development and progression of AD. This review explores mitochondria-lysosome communication through MCSs, the tethering proteins and their functions in the cell, discussing the methodological challenges in measuring the structure and dynamics of contacts, and the potential role of altered mitochondria-lysosome communication in the context of organelle dysfunction related to neuron impairment in AD pathogenesis. The different abundance of the tethering proteins was considered in healthy physiological and in AD-related conditions to assess the possible organelle communication dysregulation and the subsequent cellular function alterations, and to evaluate the role of mitochondria-lysosome MCSs in the pathogenesis of this disorder.

## 1. Introduction

Alzheimer’s disease (AD) is a neurodegenerative pathology characterized by a progressive loss of the neuronal system and dementia impacting various facets of an individual’s life and personality, encompassing physical, psychological, social, and existential suffering [1]. As the world population continues to grow and age, the increasing incidence of neurological diseases represents a major health issue. Despite the remarkable progress achieved in modern medicine over the past years, the effective diagnosis and treatment of AD continues to confront significant challenges [2].

Neuronal damage and death are the main biological characteristics of AD. Neuronal integrity and health are strictly related to the homeostasis of intracellular organelles. Indeed, maintenance of cellular functions, responses to changes, and dysregulation are closely linked to the coordination and communication of intracellular organelles. In the last few decades, several findings have highlighted that the exchange of material and signals between organelles does not only occur through vesicular trafficking and diffusion across the cytoplasm, but also through the establishment of direct communication between different organelle types via the formation of functional contacts without organelle membrane fusion to allow non-vesicular transfer of small molecules and ions [3]. The physical connection points are represented by proteinaceous tethers and are referred to as membrane contact sites (MCSs), through which organelles exchange materials such as metabolites, lipids, and ions [4,5]. MCSs were described between almost every type of subcellular organelle, such as mitochondria, endoplasmic reticulum (ER), peroxisomes, Golgi apparatus, endosomes, and lysosomes. Also, they were discovered between organelles and lipid droplets (LDs) [6], and within organelle membranes, such as the mitochondrial contact site and cristae organizing system (MICOS) in which inner and outer membranes of mitochondria form contact sites contributing to the maintenance of mitochondrial and respiratory chain architecture, cristae structure, import of protein and metabolism of lipids [7,8]. Among all, membrane contacts between ER and mitochondria are the most studied and characterized [9,10]. Inter-organelle interactions were also referred to as “organelle contactome” to define the entire set of organelle contact sites within a cell at any specific time or under certain metabolic and nutritional conditions, as well as their dynamics in both physiological and pathological states. MCSs are distinguished by an intermembrane gap ranging from 5 to 50 nm and the presence of tethering proteins or lipids that determine their specificity [11]. They are increasingly viewed as critical points where nutrients, metabolites, ions, and lipids are finely regulated to maintain cellular stability. Indeed, changes in the physiological tethering of organelles result in variations in the exchange of materials and cellular dysfunctions, which may represent a pathological condition. Hence, their molecular characterization has recently gained major interest as a potential target for therapeutic intervention [12,13,14,15].

In this context, mitochondria and lysosomes are closely interconnected, both functionally and physically. Abnormalities in lysosomes and/or mitochondria have been reported in several human pathologies, including neurodegenerative diseases. Contact sites between these organelles have been described by Wong et al. in 2018 [16] and have become a focus of research. In general, dysregulation of lysosomal and mitochondrial communication at MCS is increasingly linked to aging and age-related disorders, in particular neurodegenerative disease [17]. Indeed, impaired formation and function of mitochondria-lysosome contacts have already been reported in experimental models of genetic mutations associated with different neurodegenerative diseases [18], including Charcot–Marie–Tooth disease [19,20,21], Parkinson’s disease [22,23,24], and lysosomal storage disorders [25,26]. The effect of the alterations in the levels of tethering proteins that may be responsible for MCSs impairment, compromising the organelle network and metabolite exchange, remains largely unexplored. The lack of information is further hindered by the absence of techniques to easily evaluate organelle proximity and contact site dynamics in living cells and *in vivo*, making research in this field extremely challenging.

In AD, numerous studies reported the centrality of both lysosomal and mitochondrial deficits. In this review, attention was paid to their contacts, raising the question of whether and how disrupted crosstalk between these two organelles involves mitochondria-lysosome contact sites as contributors to disease pathogenesis and how they could be used as a therapeutic target. Hence, numerous questions are yet to be addressed in this field. For example, how do organelle contacts become functionally compromised? Are there specific roles of mitochondria–lysosome contacts in AD neuron damage and death? Is it possible to target MCSs to restore a correct mitochondria–lysosome communication, and could this partially reverse the progression of pathological conditions? The advances in understanding in this field are rapid and exciting; however, further technological and methodological advancements are necessary. Here, we focus on mitochondrial and lysosomal dysfunction in AD, as well as their contact sites, tethers, regulation, functions, and current techniques for their investigation, to understand their potential role in AD neuronal degeneration.

## 2. Mitochondria–Lysosome Contacts Formation and Dynamics

In eukaryotic cells, the concept of mitochondria as the “powerhouse of the cell” and lysosomes as the “recycling center” is obsolete. The evolution of knowledge about these pivotal organelles and their crosstalk has highlighted them as essential regulatory and signaling centers, capable of generating adaptive responses in response to changes in the cell’s metabolic and redox status, thereby helping to maintain cellular homeostasis [27,28,29]. To fulfill their roles within the cell, mitochondria and lysosomes communicate through physical interactions mediated by specialized MCSs [16], enabling bidirectional crosstalk and the establishment of a network. Mitochondria–lysosome contacts are notably different from the pathways involved in mitochondrial degradation by lysosomes. Interestingly, mitochondria but not mitochondrial-derived vesicles establish these contacts [30]. Furthermore, there is no bulk transfer of intermembrane space or matrix mitochondrial proteins into lysosomes. Likewise, MCSs do not allow the transfer of lysosomal lumen contents into mitochondria, indicating that the transfer of entire organelles does not take place through this pathway [16,18].

**Figure 1:**
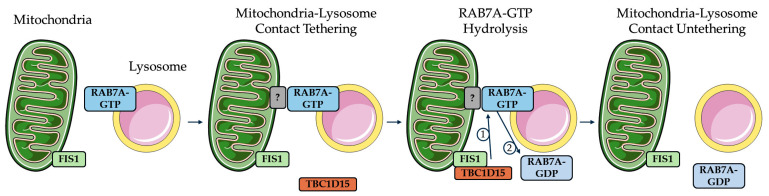
Regulation of contact site formation and tethering mediated by RAB7A. Active GTP-bound RAB7A (RAB7A-GTP), located on the lysosomal membrane, promotes contact formation with mitochondria to tether the lysosome to the mitochondria. Different RAB7A interactor proteins or complexes (gray box with question mark) were reported, but it is not clear whether RAB7A can directly bind mitochondria. FIS1, located on the outer mitochondrial membrane, recruited cytosolic TBC1D15 (RAB7 GAP). The latter drives the hydrolysis of RAB7A-GTP from an active GTP-bound state to an inactive GDP-bound state (1), making RAB7A-GDP not able to bind its effectors on the mitochondrial membrane at MCSs and to stay located on the lysosomal membrane (2), leading to the untethering of the contact. The gray box with a question mark represents the effector proteins, protein complexes, or no protein that may tether the lysosome to the mitochondria via active GTP-bound RAB7A. Image adapted from Servier Medical Art https://smart.servier.com/ (accessed on 25 July 2025), licensed under CC BY 4.0 https://creativecommons.org/licenses/by/4.0/ (accessed on 25 July 2025).

The dynamic formation of mitochondria–lysosome contacts, described by Wong et al., in 2018 [16], has rapidly become a research focus due to their importance in maintaining cellular homeostasis, metabolic signaling, and mitochondrial quality control. A role of these contacts was reported in several cellular processes, in particular mitochondrial fission and dynamics, and in the exchange of different metabolites and ions, like cholesterol, amino acids, calcium, and iron [16,25,26,31,32,33,34,35]. In parallel, dysregulation of the interactions between these organelles has been linked to diseases, particularly neurodegenerative disorders [17]. The establishment and dynamics of contacts between mitochondria and lysosomes are carefully controlled by several proteins located on the membranes of both organelles. In particular, the interaction for the establishment of the contact sites is dependent on the tethering proteins. RAB7A, a small GTPase that plays multiple roles in the late endocytic pathway, regulates several processes such as the trafficking from early endosomes to lysosomes, the maturation of early endosomes, lysosomal biogenesis, and the clustering and fusion of late endosomes and lysosomes in the perinuclear region [36,37]. GTP-bound RAB7A localizes to lysosomes and regulates the dynamics of aggregation and disaggregation by interacting with potential effector proteins on mitochondria [16,38]. Indeed, the formation of contacts is promoted by active GTP-bound RAB7A while subsequent hydrolysis from a GTP- to GDP-bound state enables the untethering of organelles at contact sites. The hydrolysis at the mitochondria–lysosome contact site was driven by the RAB7A GTPase-activating protein TBC1D15, which binds the outer mitochondrial membrane protein mitochondrial fission 1 (FIS1) [39,40] (Figure 1).

**Figure 2:**
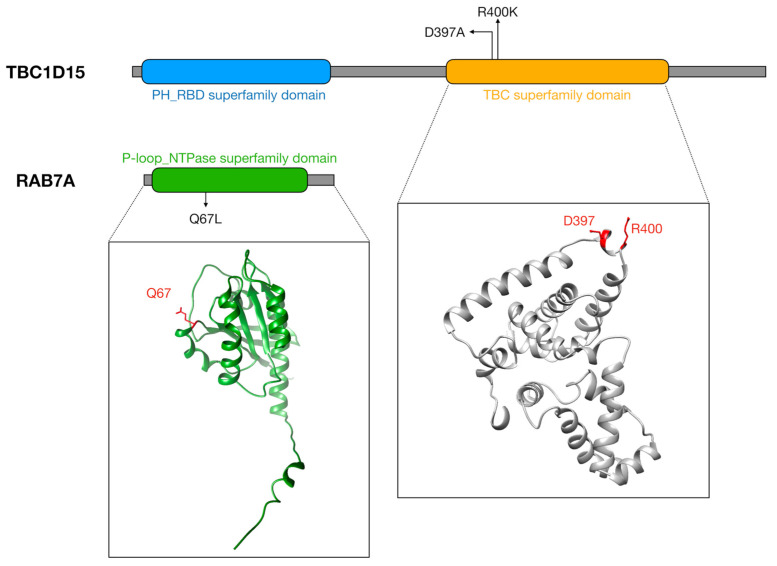
Domain organization and structural localization of mutations in TBC1D15 and RAB7A. Schematic representation of the domain structure of TBC1D15, showing the PH_RBD superfamily domain and the TBC superfamily domain. The AlphaFold [41,42] model of the TBC superfamily domain highlights the positions of mutations D397 and R400 (red) within the TBC superfamily domain. Schematic representation of the domain structure of RAB7A, with the P-loop_NTPase superfamily domain highlighted in green. The AlphaFold [41,42] model of RAB7A highlights the positions of Q67 mutation (red) within the protein structure.

This was demonstrated with the use of the constitutively active RAB7A(Q67L)-GTP mutant (Figure 2), a non-natural variant showing inhibited intrinsic GTP hydrolysis activity, leading to a higher percentage of lysosomes in contact with mitochondria and extended tethering duration compared to wild-type RAB7A [16]. Concordantly, in the axons of retinal ganglion cells of Xenopus, RAB7A(Q67L)-GTP mutant resulted in extended contact durations between mitochondria and late endosomes/lysosomes [19]. Moreover, the use of TBC1D15 GAP-domain mutants (D397A, R400K, Figure 2) or the mutant FIS1(LA), which cannot bind TBC1D15 to mitochondria, to inhibit RAB7A GTP hydrolysis resulted in inefficient untethering and extended durations of contact between mitochondria and lysosomes [16].

RAB7A interactor proteins or complexes on the mitochondrial outer membrane were reported, but it is not yet clear whether RAB7A can also bind directly to mitochondria. In HeLa cells, the interaction between RAB7A and VPS13A, as well as the impairment of the endolysosomal pathway in the absence of the latter, has been reported. Due to the subcellular localization of VPS13A to mitochondria, the authors suggested a role for VPS13A in the mitochondria–lysosome communication [43]. Neurons with mutated Parkin showed reduced mitochondria–lysosome MCSs due to the destabilization of active RAB7A, an accumulation of aminoacids in lysosomes, and mitochondrial deficit, demonstrating a role of Parkin in mitochondria–lysosome contacts and amino acid homeostasis [35].

**Figure 3:**
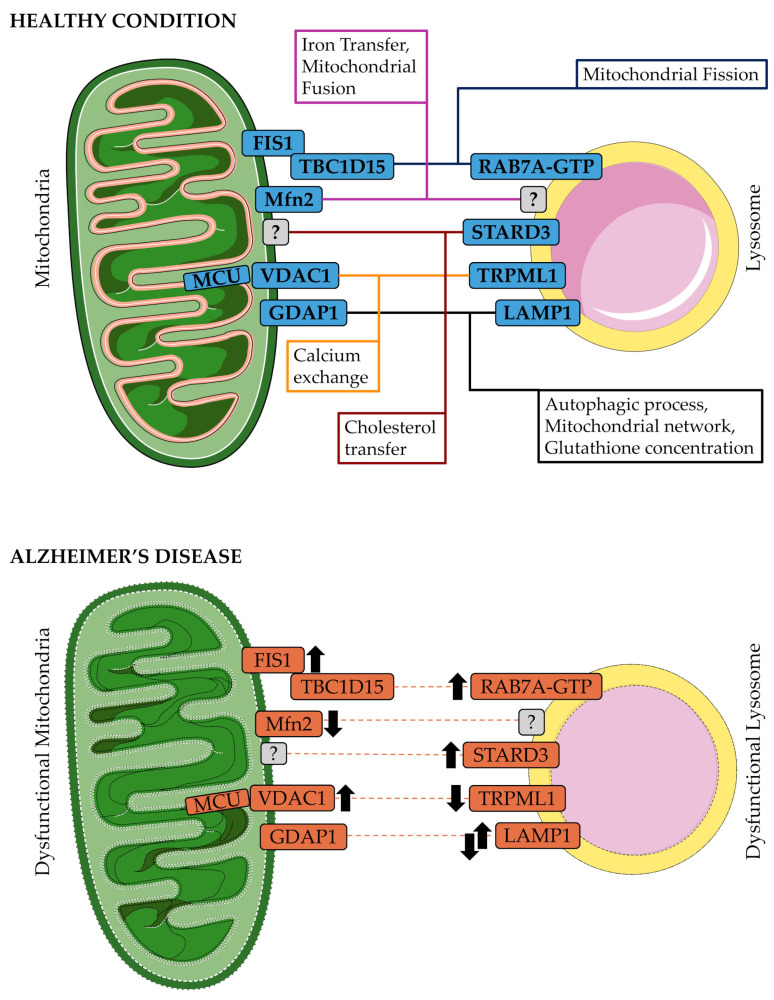
Interactions at mitochondria–lysosome membrane contact sites (MCSs). Structure of mitochondrial lysosome MCSs, comprising the mitochondrial outer membrane, the lysosomal membrane, the tethering proteins and connectors. Mitochondria–lysosome contact sites are responsible for different cellular processes (colored boxes) fundamental to maintaining cellular homeostasis and the exchange of metabolites in healthy cells. Each of these processes is mediated by different proteins forming an MCS. In the context of AD, the dysfunction of mitochondria and lysosomes may alter this balance and the processes regulated by MCSs. In the upper part of the figure, the principal actors of MCSs and cellular processes in physiological healthy conditions are reported. In the lower part, the dysregulation of the protein levels in AD was reported to evaluate a potential alteration in mitochondria-lysosome MCSs. Grey boxes with a question mark indicate an unknown binding interactor for Mfn2 at the lysosome membrane and for STARD3 at the mitochondrial membrane. Black up arrows indicate an increase in the interactor level in AD, while black arrows pointing down indicate reduced levels of the interactor in AD. The black up and down arrows indicate contrasting information regarding the levels of LAMP1 protein in AD. The protein boxes without any black arrow next to them (TBC1D15 and GDAP1) indicate that, to the best of our knowledge, no information was available about the alteration of their levels in AD cells. Image adapted from Servier Medical Art https://smart.servier.com/, (accessed on 25 July 2025) licensed under CC BY 4.0 https://creativecommons.org/licenses/by/4.0/ (accessed on 25 July 2025).

More recently, other factors involved in regulating mitochondria–lysosome contact sites have been proposed. The glutathione S-transferase GDAP1, located on the outer mitochondrial membrane, is reported to be involved in the autophagic process and in the maturation of lysosomes. Its depletion affects LC3, PI3P, and the autophagosome biogenesis, causes giant lysosomes, a delay in the autophagic lysosome reformation, and the activation of a regulator of lysosomal biogenesis, the transcription factor EB (TFEB). Furthermore, GDAP1 interacts with LAMP1, a lysosomal membrane protein, forming MCS between the two organelles (Figure 3). Based on co-immunoprecipitation and proximity ligation assays conducted in human neuroblastoma SHSY5Y cells and in soma and axons of *in vitro* mouse embryonic motor neurons, when GDAP1 is knocked down, there is an increase in the distance between mitochondria and lysosomes, a reduction in the number of contact points between the two organelles, and a decrease in the duration of these contacts, resulting in mitochondrial network alterations and a decrease in cellular glutathione (GSH) levels. These findings suggest that GDAP1-LAMP1 may be involved in the tethering of mitochondria and lysosomes [20].

The interactions between mitochondria and lysosomes are influenced by various other tethering proteins and pathways (Figure 3), which are only partially described and characterized to date, depending on the specific context, which could involve a wider array of inter-organelle contact sites. Further investigations are needed to determine the existence of other protein complexes that also play a role in modulating the dynamics of contact tethering, acting together or in addition to the regulation of mitochondria–lysosome interactions by RAB7A GTP hydrolysis.

## 3. Functional Roles for Mitochondria–Lysosome Contacts in Regulating Cell Homeostasis

Functionally, mitochondria–lysosome contacts are crucial for managing the dynamics of both mitochondria and lysosomes and for maintaining metabolite homeostasis through the two-way exchange of calcium, cholesterol, iron, and other metabolites [16,26,31,33,44,45,46]. Enhancing our understanding of the complex interactions between organelles will be essential for developing new therapeutic strategies aimed at correcting defects in the exchange of cell components, such as lipids and calcium, and at addressing imbalances associated with various disease conditions.

### 3.1. Mitochondrial Fission and Network

Mitochondria–lysosome contacts were described as fundamental in mitochondrial fission, where RAB7A GTPase contacts identify the point of mitochondrial fission, enabling the regulation of mitochondrial networks through lysosomes (Figure 3). Alterations in the formation and maintenance of these MCSs can influence mitochondrial dynamics, fission and fusion, and increase mitochondrial autophagy, a characteristic of various human diseases [16]. Furthermore, in monkey and mouse cells, mitochondria–lysosome MCSs were associated with a specific type of fission mediated by DRP1 and involving division at the periphery that allows damaged components to be released as smaller mitochondria destined for mitophagy. In this model, peripheral fission is initiated by lysosomal contact and is controlled by the mitochondrial protein FIS1, which recruits the tethering molecule TBC1D15 to form a mitochondrial-lysosomal contact. This process was observed in 92% of peripheral fissions [44]. FIS1 is also one of the receptors on the mitochondrial surface of DRP1, the key protein in mitochondrial fission and apoptosis processes [47]. In addition, GDAP1 interaction with LAMP1 influences the mitochondrial network (Figure 3). Knockdown of GDAP1 reduces the number and the duration of mitochondria–lysosome MCSs, leading to alterations in the mitochondrial network and a decrease in cellular GSH [20].

### 3.2. Calcium Exchange Between Mitochondria and Lysosomes

MCSs were reported to be involved in the direct exchange of Calcium (Ca^2+^) ions from lysosomes to mitochondria, thereby regulating the intracellular concentration and dynamics. The concentration of Ca^2+^ directly influences cellular processes like apoptosis, mitochondrial metabolism, and ATP production. Mitochondria are the key regulators of calcium ion levels, and their functionality is linked to the uptake of intracellular Ca^2+^. Transient receptor potential mucin channel 1 (TRPML1), localized on late endosomal and lysosomal membranes, is recognized as a significant site for the interaction between lysosomes and mitochondria, facilitating the regulation of ion concentration between these organelles and allowing the direct transfer of Ca^2+^ from lysosomes into the mitochondria, regulating their functions [26,48]. At the mitochondrial site, voltage-dependent anion channel 1 (VDAC1) on the outer membrane and the mitochondrial calcium uniporter (MCU) [49,50], located on the inner membrane, modulate the transfer of Ca^2+^ from lysosomes to mitochondria at their contact sites (Figure 3). The use of agonist ML-SA1, which activates TRPML1, causes an increase in Ca^2+^ levels selectively in mitochondria that form contact sites with lysosomes. On the other hand, the expression of a negative TRPML1 mutant, which generates a defective pore, reduced the Ca^2+^ influx in mitochondria [26]. Taken together, the dynamics of mitochondria–lysosome tethering can significantly influence the concentration of calcium in both mitochondria and lysosomes.

### 3.3. Iron Transfer

MCSs are essential for managing the labile iron pool, specifically for the transfer, as the lysosome acts as a storage site for iron (Fe^2+^). The mechanism for iron exchange is still debated; a “kiss-and-run” mechanism similar to the one that promotes Fe^2+^ transfer from early endosomes to mitochondria was proposed [51,52]. An actor in this exchange via mitochondria–lysosome contacts may be TRPML1 due to its permeability [53]. Another protein involved may be the transferrin receptor-2 (TfR2), which is responsible for delivering transferrin. A deficiency in TfR2 results in aberrant mitochondrial morphology, characterized by smaller size and less heme content, in erythroid progenitors [46,54]. Expression of TfR2 was related to Mitofusin 2 (Mfn2), a transmembrane GTPase located on the outer membrane of mitochondria and required in mitochondrial-lysosomal contacts and regulation [46]. The knockdown of Mfn2 caused a decrease in the number of contacts and a reduction in heme content in primary human erythroid progenitors [46,55], making Mfn2 a mitochondria–lysosome MCS protein (Figure 3). Moreover, Mfn2 is a key player in multiple biological processes in both normal and disease states. It is fundamental in the regulation of mitochondrial-related activities such as fusion, trafficking, turnover, apoptosis, and contacts with other organelles [56].

In a recent preprint 3-hydroxybutyrate dehydrogenase 2 (BDH2) protein, a dehydrogenase/reductase family member, was suggested to be a key effector in organelles Fe^2+^ redistribution, localizing at the mitochondria–lysosome contacts, transferring iron that endorses mitochondrial OXPHOS and ATP production which is then utilized by lysosomes to maintain a low pH through the activity of V-ATPase for lysosomal acidification in melanoma cell lines. In particular, BDH2 was found to produce 2,5-dihydroxybenzoic acid (2,5-DHBA), a compound that plays a crucial role in transporting iron from lysosomes to mitochondria [57]. The acidic lysosomal pH is fundamental for the Fe^2+^ transfer to mitochondria. The impairment of pH-dependent mechanisms includes the functionality of TfR and the lysosomal iron transporters [58,59], but it is not clear whether it also impacts MCS formation.

### 3.4. Cholesterol Homeostasis

Contact sites between mitochondria and lysosomes have also been shown to play a crucial role in regulating cholesterol homeostasis, which is fundamental for membrane integrity and acts as a precursor for several types of signaling molecules. Niemann Pick C2 (NPC2) protein, in lysosomes, was reported to regulate the movement of cholesterol from lysosomes to mitochondria, particularly at contact points between the two organelles [33]. In addition, the endoplasmic reticulum is the key organelle for cholesterol production and calcium storage. Indeed, the crosstalk between organelles may involve more than one MCS. For example, organelle communication between the ER and mitochondria is essential for cholesterol and calcium homeostasis in the cell. The formation of a three-way contact, ER–mitochondria–lysosome, highlights the intricate interactions among these organelles, which play a crucial role in coordinating and integrating cellular responses to various demands and stressors [32,60]. Hence, Niemann Pick C1 (NPC1) protein, located on the lysosomal membrane, is suggested to facilitate the transfer of cholesterol between lysosomes and the ER by interacting with the ER sterol-binding protein Gramd1b. It was reported that the loss of NPC1 decreases the ER’s interactions with endocytic organelles, leading to cholesterol accumulation in lysosomes and an increase in contacts between mitochondria and lysosomes through the action of the sterol transfer protein StAR-related lipid transfer domain-3 (STARD3) [25]. STARD3 (also known as metastatic lymph node 64 protein MLN64), localized in late endosomes and lysosomes, has been linked to the transport of LDL-cholesterol to mitochondria [61] (Figure 3), and its depletion leads to a high reduction in mitochondria–lysosome contacts [25].

### 3.5. Homeostasis of Other Lipids

Moreover, lipid homeostasis may also be influenced by the mitochondria–lysosome MCS. ORP1L, located on the endolysosomal membrane, plays a role in transporting lipid PI(4)P from lysosomes to mitochondria at MCSs [32]. In yeast, the vacuole and mitochondria patch (vCLAMP) has been demonstrated to facilitate the transfer of phospholipids such as phosphatidylserine and phosphatidylcholine [62] involving other protein complexes, such as mitochondrial Tom40/VPS39/vacuolar RAB GTPase Ypt7 [63], and mitochondrial MCP1/Vps13 in conjunction with vacuolar Ypt35 [64]. Furthermore, dysregulation of the ceramide pathway can lead to extended durations of MCSs in neurons, underscoring the intricate link between this pathway and the regulation of various metabolites [24].

### 3.6. Localization of Protein Synthesis

Lastly, interactions between mitochondria and late endosomes/lysosomes were described in the localization of axonal mRNAs to specific subcellular sites for translation. Specifically, ribonucleoprotein particles (RNPs) were observed to associate with motile RAB7A-positive late endosomes along retinal ganglion cell (RGC) axons of Xenopus. RNP-bearing RAB7A-positive late endosomes frequently dock at mitochondria, creating hotspots for de novo synthesis of proteins involved in maintaining mitochondrial integrity and promoting axon survival. Disruption of RAB7A function using RAB7A mutants, including those linked to Charcot–Marie–Tooth type 2B neuropathy, leads to significantly reduced axonal local protein synthesis, mitochondrial dysfunction, and loss of axon integrity. Therefore, the localized presence of RNPs and the synthesis of nascent proteins at specific neuronal locations suggest that mitochondria–lysosome interactions might play additional specialized functions in supporting neuronal homeostasis [19].

### 3.7. Regulation of Autophagy Pathways

Mitochondria–lysosome contacts have distinct functions compared to mitochondria–lysosome interaction associated with lysosomal degradation processes such as autophagy/mitophagy or mitochondrial-derived vesicles [30,65]. GDAP1, which forms a mitochondria lysosome contact with LAMP1, is an important actor in the autophagic process. Although mitochondria–lysosome contacts are different from pathways involved in mitochondrial degradation by lysosomes (see Section 2), a subtype of these contacts defined by their short-range interaction (~4 nm) was suggested to be co-regulated by autophagy pathways [34]. Still, their role remains to be fully elucidated.

**Figure 4:**
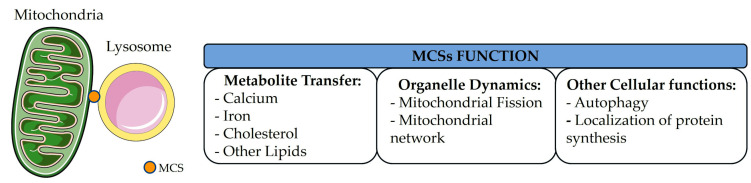
Main functions of mitochondria–lysosome MCSs. These contacts are important for Ca^2+^ dynamics, iron metabolism, cholesterol and other lipids exchange. They are involved in organelle dynamics, especially in mitochondrial fission. A role for these contacts has been proposed in other related cellular functions, such as autophagy and protein synthesis. MCS: membrane contact site. Image adapted from Servier Medical Art https://smart.servier.com/ (accessed on 25 July 2025), licensed under CC BY 4.0 https://creativecommons.org/licenses/by/4.0/ (accessed on 25 July 2025).

A schematic summary of mitochondria–lysosome MCSs function is reported in Figure 4.

## 4. Methodologies to Study Mitochondria–Lysosome Communication and Techniques for Probing Interactions

The study of mitochondria–lysosome interaction is challenging because of the highly dynamic and transient nature of contacts, which makes the detection very difficult. Two critical parameters need to be taken into consideration: the distance between the organelles (~10 nm) and the duration of their closeness (>10 s) [16,34]. Various methods have been used for characterizing MCS; actually, the gold standards are considered advanced microscopy techniques. Most of these methodologies have been adapted to specifically evaluate mitochondria–lysosome contact numbers, duration, structure, dynamics, and function, yielding significant insights. However, each methodology comes with its own set of limitations, so the use of several complementary techniques can enhance the reliability of the findings [53,66].

To visualize the ultrastructure of MCSs and the surrounding cellular context, Electron microscopy (EM) techniques are used. The most important limitation of these approaches is the need to fix the sample, which makes it impossible to capture the dynamic nature of organelle contact sites in living cells. To explore the ultrastructural architecture of MCSs and pinpoint the proteins in the organelles’ interfaces, Transmission Electron Microscopy (TEM) analysis, which requires cell fixation, was performed [67] together with biochemical staining methods including horseradish peroxidase (HRP), immunogold labeling, or APEX2 tags [53]. The latter are broadly applicable genetic tags, mainly used in fusion constructs that generate contrast on a specific labeled membrane protein to which they are fused [68]. Correlative Light and Electron Microscopy (CLEM) approaches, a combination of fluorescence microscopy with EM in which the latter provides high-resolution information, while the former highlights the regions of interest, offer especially effective means for investigating the MCSs [69,70]. Indeed, a recently developed protocol in which cells were co-transfected with plasmids Mito-mEosEM and TMEM192-V5-APEX2 to label mitochondria and lysosomes, respectively, was developed for fixed cells. The resulting CLEM images highlighted the changes in organelle interactions under stress conditions. Indeed, the treatment with bafilomycin to inhibit lysosomal function showed an increase in contacts, while the use of U18666A to inhibit the exchange of cholesterol from lysosomes led to the clustering of mitochondria around lysosomes, making this approach a powerful tool for studying organelle contact in both healthy and pathological conditions [70]. In recent years, Cryo-EM has analyzed samples in vitrified ice at cryogenic temperatures, providing a tool to visualize the 3D structures of proteins assembled on membranes *in vitro* and in their native cellular state in physiological solutions. Nevertheless, there is a notable lack of cryo-EM published studies on MCSs, highlighting that its application in this field of research remains relatively limited and hardly accessible [71,72].

On the other hand, Super-Resolution Fluorescent Microscopy techniques are extensively utilized for the in-depth characterization of MCS. Due to the average distance between mitochondria and lysosomes of around 10 nm, the light diffraction limits of conventional fluorescence microscopy are not appropriate to obtain a structural image. These techniques utilize various approaches to overcome the limitations imposed by light diffraction, such as alterations in the excitation and emission processes of fluorescent molecules. Among the various techniques available, MCS has mainly been explored through four key methodological approaches: Structured Illumination Microscopy (SIM), Total Internal Reflection Fluorescence Microscopy (TIRFM), Stimulated Emission Depletion (STED) microscopy, Single Molecule Localization Microscopy (SMLM), the latter including Photo-Activated Localization Microscopy (PALM), STochastic Optical Reconstruction Microscopy (STORM) and Points Accumulation for Imaging in Nanoscale Topography (PAINT) [73,74,75]. Resolution of approximately 10–20 nm in living cells also permits the evaluation of contact durations and dynamics. Mitochondria and lysosomes could be labeled with specific dyes such as MitoTracker or tetramethylrhodamine methyl ester (TMRM) and LysoTracker, respectively, or fluorescent constructs with lysosomal or mitochondrial membrane proteins and a genetically encoded tag such as GFP to facilitate the live-cell imaging of contact dynamics. The combination of fluorescent dyes and time-lapse microscopy enables the measurement of frequency, duration, and movement of mitochondria–lysosome contacts [53]. Another method that was used is the Fluorescence Resonance Energy Transfer (FRET). Using the right FRET pairs (donor and acceptor fluorophores), it was possible to identify the close proximities (1–10 nm) between organelles in live cells. Outer mitochondrial membranes and lysosomal membrane proteins were tagged with fluorophores, and FRET allows for the detection of energy transfer between the tagged proteins associated with the respective organelles, which indicates the formation of a contact site. Wong et colleagues used TOM20-Venus (outer mitochondrial membrane) and LAMP1-mTurquoise2 (lysosomal membrane) to confirm the formation of contact in living cells [16]. Moreover, a split-GFP-based contact site sensor, which was designed to fluoresce when organelles are in proximity [76], has been used and implemented by Giamogante et al. to investigate the tethering between lysosomes and mitochondria *in vitro* and *in vivo*. With this system, named SPLICSS/L-P2ALY–MT, two types of mitochondria–lysosome contact sites in human cells were described, short-(~4  nm) and long-range interface (~10 nm), each of which showed a different response in the presence of specific stimuli given by changes in the levels of proteins involved in tethering and untethering [34]. With these experiments, they highlight that only the short-range MCSs were dependent on the RAB7A/TBC1D15 pathway and that these contacts may be co-regulated by autophagy pathways [34].

Taken together, MCSs between mitochondria and lysosomes were first observed by STORM [52] and successively by EM, and FRET [16]. A summary of the most relevant techniques used in previous studies on mitochondria–lysosome contacts, together with the potential benefits and limitations of each method, is reported in Table 1.

Mitochondria–lysosome contacts have an average distance between membranes of ~10 nm. Furthermore, using live confocal microscopy at high spatial and temporal resolutions, it was possible to observe that, at any given time, ~15% of lysosomes contacted a mitochondrion and that, although tethering could last as long as 13 min [73], the average duration was ~60 s [16]. Furthermore, a single lysosome could simultaneously contact multiple mitochondria, and the lysosome contacted by mitochondria could have different sizes, small (diameter <0.5 μm) and larger (diameter >1 μm) [16].

Despite recent advances, measuring the structure and dynamics of mitochondria–lysosome contacts remains challenging. In the future, advances in technologies are necessary, such as new dyes that elicit tolerable levels of phototoxicity to be used in long-term imaging and super-resolution imaging, molecular tools or drugs for the manipulation of organelle dynamics, computational methods for data processing, and large datasets of image reconstruction and analysis.

## 5. The Role of Mitochondrial and Lysosomal Dysfunctions in Alzheimer’s Disease

AD is a multifactorial neurodegenerative disorder pathologically characterized by the extracellular progressive deposition of amyloid beta (Aβ) plaque, accumulation of hyperphosphorylated tau protein, also known as neurofibrillary tangles (NFTs), within neurons, and neuronal loss. Despite intensive efforts, the main trigger of AD pathology and the mechanisms are still not fully understood [78]. A large plethora of studies have associated AD with a decline in organelle and protein homeostasis, particularly affecting mitochondria and the endosome-lysosome system [79,80,81,82].

The *e4* allele of the *APOE* (Apolipoprotein) gene is the major genetic risk factor for late-onset AD and, in neurons, has a deleterious impact on the expression and function of mitochondrial proteins. In neuroblastoma cells, the presence of apoE4 led to a reduction in the levels of mitochondrial respiratory complexes I, IV, and V. Additionally, the activity of Complex IV was diminished, which contributed to a decrease in mitochondrial respiratory capacity [83]. ApoE4 produces an imbalance in mitochondrial dynamics; it has been linked to abnormal fusion and fission and reduced mitophagy in the hippocampus of ApoE4-transgenic mice [84] and to decreased fission and impaired parkin-mediated mitophagy in APOE4 astrocytes [85]. Additionally, mutations in mitochondrial DNA (mtDNA) are found more frequently in the brains of AD patients compared to age-matched controls, potentially contributing to the mitochondrial dysfunction observed in these patients [86,87]. Rarer point mutations in the *MT-RNR1* and *MT-TR* genes in homoplasmy have been identified in AD, and the mtDNA copy number was found to be lower in AD patients than in controls [88]. Furthermore, point mutations in mtDNA that encode for subunits of Complex IV have been linked to diminished cytochrome c oxidase (COX) activity, which may play a role in the progression of AD [89].

A considerable amount of evidence indicates that dysfunctional mitochondria may be central to the development of AD. In the brain, where neurons require a significant amount of energy to support synaptic activity and plasticity, mitochondrial alterations are among the earliest noticeable changes. Indeed, to support neuronal activity, healthy functional mitochondria are necessary to satisfy the energy demand to perform critical functions and to protect neurons by reducing oxidative damage associated with mitochondrial dysfunction [90]. Longitudinal studies indicate that mitochondrial dysfunction occurs before the Aβ accumulation and tau deposit, suggesting its role as an early event in the development of AD [91]. Mitochondrial dysfunction compromises neural homeostasis and survival, leading to early neuronal death and the emergence of AD symptoms [90]. The impairment is associated with different interrelated factors such as metabolic imbalances, elevated oxidative stress, disruption of calcium homeostasis, and compromised mitochondrial quality control [92,93]. Furthermore, mitochondrial dysfunction is closely connected to key characteristics of AD, such as Aβ plaque accumulation and tau hyperphosphorylation [94]. Taken together, mitochondria are considered so important in AD development that, alongside the initial Amyloid Cascade Theory, the Mitochondrial Cascade Hypothesis has been formulated, proposing that they mediate, drive, or contribute to a variety of AD-caused alterations [95,96,97]. Mitochondrial dysfunctions in AD were recently reviewed in [92,98,99].

On the other hand, the autophagy-lysosomal pathway is essential for the degradation and recycling of dysfunctional organelles and aggregate amyloid proteins associated with AD for maintaining cellular homeostasis. Lysosomes are considered the primary site of intracellular toxic Aβ accumulation, making them a potential primary root for multi-organellar dysfunctions. Alterations of the lysosomal system are considered a feature of AD [100], and several studies have highlighted abnormal autophagic activity, impaired lysosomal acidification, and inefficient cellular degradation, which could impact the clearance of amyloidogenic or aggregate-prone proteins involved in disease progression [101,102]. Moreover, an elevated number of autophagosomes was found in the brain tissues of AD individuals, which may be due to reduced lysosomal function or impaired autophagosome-lysosomal fusion [103,104]. Lysosomal dysfunction has been linked to early phases of AD, in the process of accumulation of toxic protein aggregates, prior to amyloid plaque formation and to the advanced stages of this disease characterized by neuronal death and toxic protein deposition [105,106], indicating that such dysfunction could play a critical role in the buildup of amyloid plaques and the overall development of AD. Furthermore, it was observed that brain tissues from AD patients are notably characterized by the presence of Aβ plaques rich in lysosomal proteins [107,108], and genetic models for AD accumulating Aβ or tau aggregates, show endosome–lysosomal dysfunction [82,109,110]. The causal link for the accumulation of amyloid and lysosomal damage remains unclear. It was hypothesized that the endocytosis of extracellular protein oligomers increased in AD, leading to lysosomal damage, which in turn promotes the seeding of aggregates and neuronal death [111,112,113]. More recent studies have shown in AD neurons the presence of intrinsically perforated endosome-lysosomes, which may support the seeding of cytoplasmic aggregates [114,115]. For an exhaustive recent literature review of lysosomal dysfunction in AD, see [116,117].

Taken together, although the cause of their onset is not yet known, both mitochondrial and lysosomal dysfunction are well-characterized in AD.

## 6. Potential Role of Mitochondria–Lysosome Contact Sites in Alzheimer’s Disease

In the context of AD, the role and involvement of mitochondria–lysosome MCSs in neuronal degeneration remain largely unexplored. Here, we have considered some aspects and cellular functions associated with AD pathogenesis that were reported as relevant in mitochondria–lysosome MCSs, as well as some proteins with a distinctive role observed in both AD and MCSs, with a focus on tethering proteins reported in Section 3. Both mitochondrial and lysosomal dysfunctions were reported as early events in AD-affected brains. Although the initial trigger remains unclear, the functional mutual dependence of the two organelles, as reported by evidence obtained from cells with mitochondrial alterations or defective lysosomes, may suggest a role for MCSs and a link between the impairments. Therefore, it is possible to hypothesize that in the case of a mitochondrial deficit in neurons due to aging, the communication between organelles becomes no longer functional, the exchange of molecules is altered, with a consequent loss of cellular homeostasis. This may contribute to lysosomal impairment. Hence, mitochondrial dysfunction has a harmful impact on lysosomes’ functionality, as observed in various cell types both *in vitro* and *in vivo* [118,119,120,121,122]. Apparently, this is not directly due to reduced ATP availability. Indeed, how the lysosomal compartment functions are dysregulated by mitochondrial dysfunction, is still not fully understood. The bioenergetic deficit and the consequent decrease in ATP concentration have been reported as an essential factor. Still, other actors may be involved with a direct effect on lysosomal function, such as the mitochondria–lysosome tethering proteins [118,119,120,121,122]. For instance, the impairment of mitochondrial function due to the deletion of fundamental mitochondrial proteins such as AIF, OPA1, or PINK1 or the use of inhibitors of the electron transport chain leads to lysosomal dysfunction, evidenced by large LAMP1-positive lysosomal vacuoles, which become nonacidic and lose their hydrolytic activity [118]. On the other hand, in yeast, the impaired vacuole acidification was reported to limit mitochondrial function [123]. Functionally, the reduced ability of lysosomes to degrade materials has significant implications for mitochondrial quality control (MQC), as mitophagy, the process responsible for the degradation of damaged mitochondria, relies on functioning lysosomes for its effectiveness [123]. Accordingly, studies in fibroblasts from Down syndrome patients, predisposed to early-onset AD due to the extra chromosome 21, which carries the gene for amyloid precursor protein (*APP*), accumulate the β-cleaved carboxy-terminal fragment of APP that impairs lysosomal acidification and functionality by inhibiting the V-ATPase, contributing to ineffective mitophagy [124]. Taken together, it is possible to hypothesize that during the early onset of disease, one of the two organelles may lose its normal function, affecting cellular homeostasis. In this context, the mitochondria–lysosome contacts may be reduced or altered, causing an alteration in organelle balance, in their functions, and a loss of communication. On the other hand, organelle communication may also serve to amplify the dysfunction from one organelle to the others in the cell.

In AD, an imbalance between mitochondrial fission and fusion was reported, with a significant shift toward fission. Hence, the affected neurons possess dysfunctional mitochondria that fail to satisfy the cell’s metabolic requirements [125]. In particular, increased expression of DRP1 and FIS1 and decreased expression of Mfn1, Mfn2, Opa1 and Tomm40 were reported in APP transgenic mice primary hippocampal neurons and in AD brains [126,127]. Furthermore, it was found, using immunoprecipitation, immunofluorescence, and double-labeling analyses, that DRP1 interacts with intraneuronal monomeric and oligomeric forms of Aβ in patients with AD. DRP1 was mainly expressed in neurons of the frontal cortex, but it was also detected in the astrocytes and microglia of patients with AD. Additionally, intraneuronal Aβ and Aβ deposits were observed in the brains of AD patients, and monomeric and oligomeric forms of Aβ were detected in primary neurons derived from APP transgenic mice. In this context, their increased production and interaction became fundamental factors in the excessive mitochondrial fragmentation, compromising mitochondrial transport along axons and dendrites and impairing synaptic function [127]. In the context of excessive mitochondrial fission and elevated levels of DRP1 and FIS1, RAB7A levels were reported as increased in different studies: in the basal forebrain, frontal cortex and hippocampus of mild cognitive impairment and AD patients, high levels of RAB7A were observed compared to controls [128,129,130]. Moreover, in nucleus basalis neurons of the basal forebrain, the upregulation of *RAB7A* gene expression was shown during AD progression [131], and the cerebrospinal fluid (CSF) of AD patients exhibits an increase in RAB7A protein levels [132]. Moreover, RAB7A was reported to regulate tau secretion, and a partial co-localization of tau and RAB7A-positive structures was shown in neurons and HeLa cells. In this context, the increased expression of RAB7A observed in AD may play a role in the extracellular accumulation of pathological tau species, potentially promoting the spread of tau pathology in the brains of affected individuals [133]. The elevated mitochondrial fission observed in AD neurons and the increased levels of proteins involved in RAB7A GTPase-TBC1D15-FIS1 MCS formation and dynamics (Figure 3), which was reported to regulate fission, may indicate a higher frequency of mitochondria–lysosome MCSs as a consequence of a dysregulation in the communication between organelles through these sites.

As reported above, the levels of Mfn2 protein and mRNA, involved in mitochondria–lysosome MCSs for iron transfer, mitochondrial fusion, and other cellular processes, were decreased in AD brains and primary hippocampal neurons from APP transgenic mice [126,127]. Moreover, a decrease in phosphorylated Mfn2 levels was suggested to contribute to mitochondrial fragmentation, which in turn causes abnormalities in mitochondrial morphology and distribution in AD cybrids [134]. Additionally, in adult neurons, the deletion of Mfn2 and the consequent mitochondrial fragmentation was reported to lead to neurodegeneration via oxidative stress and neuroinflammation *in vivo* [135]. Taken together, these results indicate that Mfn2 could play a crucial role in mitochondrial dysfunction associated with AD. Decrease in Mfn2 levels may also affect the number and duration of mitochondria–lysosome MCS, leading to an imbalance in iron transfer and contributing to altered mitochondrial fusion (Figure 3). In this context, it was reported that the increase in Mfn2 expression via methylation modification in the AD model mice improved mitochondrial dysfunction and cognitive deficits, making this protein a promising therapeutic target in AD [136].

Mitophagy depends significantly on the integrity of lysosomes, which is severely compromised in AD, leading to further mitochondrial dysfunction [137]. Additionally, autophagy was found to modulate Aβ clearance and secretion and was reported as impaired in AD. In particular, in mouse models, where treatment with rapamycin or genetic modifications causes enhanced autophagy, both intracellular and extracellular deposition of Aβ in brains were reduced [138,139,140]. Aβ oligomers act as autophagic substrates and were enclosed within autophagosomes in the brain of autophagy-hyperactive AD mice [139]. On the other hand, in autophagy-deficient AD mice, extracellular Aβ plaque formation was drastically decreased while intraneuronal Aβ was markedly accumulated, indicating a compromised Aβ secretion [141]. Hence, an impairment in autophagy contributes to AD pathogenesis. In the early stages of the disease, an accumulation of autophagosomes and autolysosomes in AD patients’ brains [104] and an up-regulation of the mRNA and protein of autophagy-related and lysosomal genes in hippocampal CA1 neurons from AD patients [142] were reported. These data indicate that increased autophagy during the early stages of AD serves a protective role against stress. However, as AD progresses, autophagic flux becomes impaired [143], and the downregulation of several autophagy-related proteins was found in AD brains [144,145]. Overall, accumulating evidence points to a strong relationship between dysfunctional autophagy and the development of AD [140]. Furthermore, the accumulation of autophagic vesicles that are not able to fuse with lysosomes may exacerbate mitochondrial dysfunction. Following the different expressions of autophagic proteins, no consensus information is present in the literature regarding LAMP1 levels in AD. In CSF, neurons, and glial cells of AD patients, as well as in AD mouse models, increased levels of LAMP1 have been reported [132,146], which is associated with severely impaired lysosomal function in neurons [146]. Furthermore, amyloid plaques were found massively enriched with LAMP1, indicating an accumulation of lysosomes at these sites [147,148]. On the other hand, a reduction in the levels of LAMP1 was found in AD mice and cell models [149]. This contrasting information may highlight the evolution of the lysosomal dysfunction in the AD pathogenesis and progression, with an involvement in autophagy impairment and different LAMP1 levels in the early and late phases of disease. The impairment in the levels of LAMP1 may influence the formation of mitochondria–lysosome MCSs, mediated by GDAP1 and LAMP1 (Figure 3), contributing to alterations in the autophagic process and mitochondrial network. Furthermore, to the best of our knowledge, alteration of GDAP1 levels in AD has not been reported yet. Nonetheless, motor neurons lacking GDAP1 showed some alterations similar to neuronal AD. Indeed, these cells were shown to be prone to early degeneration, and are characterized by alterations in mitochondrial structure and network, such as increased fragmentation, in autophagy and mitophagy, and elevated levels of reactive oxygen [150].

Calcium signaling plays an essential role in regulating mitophagy, connecting mitochondrial dysfunction with neuronal damage in the context of AD pathogenesis. The interaction between tau protein and VDAC1 shows another connection between mitochondrial dysfunction and tau-related pathology [151]. VDAC1 is a crucial component of the outer mitochondrial membrane and forms contact with MCU in the inner membrane for calcium exchange and with TRPML1 on the lysosomal membrane. Functionally, VDAC1 plays a key role in regulating the transport of pyruvate and in controlling the passage of calcium ions and reactive oxygen species (ROS) into the mitochondria [151]. High levels of VDAC1 have been reported in affected regions of AD brains and cortical tissues from AD mouse models [152]. Furthermore, when tau becomes hyperphosphorylated, in the presence of Aβ peptides, it disrupts the normal functioning of VDAC1, leading to impaired mitochondrial transport processes and the promotion of apoptosis [151,153]. In this context, the formation and tethering of MCSs mediated by VDAC1 may be compromised due to a reciprocal relationship between mitochondrial dysfunction and tau hyperphosphorylation, leading to impaired mitochondrial calcium influx via the MCU, and excessive calcium accumulation, which prompts the opening of the mitochondrial permeability transition pore mPTP, releases cytochrome c, and results in apoptotic cell death [154,155]. Additionally, intracellular calcium overload can interfere with mitophagy efficiency, blocking the stabilization of PINK1 on the mitochondrial membrane and Parkin recruitment [156]. The involvement of tau pathology in MCU dysfunction further worsens mitochondrial calcium imbalance, increasing mitophagy deficits and promoting neurodegeneration [157]. Importantly, studies have demonstrated that inhibition of MCU using drugs can restore mitophagy function and alleviate AD pathology, underscoring the potential of targeting mitochondrial calcium balance as a therapeutic approach to combat neurodegenerative disorders [158]. On the other hand, TRPML1 has been identified in the cellular defects associated with AD pathogenesis for its function in the exchange of ions, such as calcium, between lysosomes and mitochondria, and for its related function as an autophagy regulator [159]. In particular, downregulation of TRPML1 was reported in APP/PSEN1 transgenic mice, a mouse model for AD research carrying mutated forms of *APP* and presenilin-1 (*PSEN1*) genes, both associated with early-onset familial disease, which is linked to a disruption in the autophagy-related PPARγ/AMPK/mTOR signaling pathway [160]. Impaired lysosomal calcium signaling, especially via TRPML1 channels, which is involved in the formation of mitochondria–lysosome MCSs and was found downregulated in AD (Figure 3), may further disrupt the autophagic flux, leading to a self-reinforcing cycle of mitochondrial and lysosomal dysfunction.

Pharmacological approaches focused on restoring lysosomal function have shown potential in restoring mitophagy in AD. For instance, supplementation with nicotinamide riboside in APP/PS1 transgenic mice has been found to enhance the expression of LC3 and PINK1, which subsequently reduces Aβ accumulation and boosts cognitive abilities [161]. Similarly, in preclinical models of AD, the mitigation of Aβ pathology and the restoration of synaptic function caused by the activation of neuronal mitophagy through compounds like nicotinamide mononucleotide (NMN) and urolithin A were observed [158].

Remarkably, the organellar interactome operates as a network to maintain cellular homeostasis. Therefore, the identified organellar dysfunctions may be associated with additional connections, especially the interactions with the endoplasmic reticulum. Concerning ER-mitochondria communication, an increase in the number and duration of contacts was observed in presenilin-mutant cells and in fibroblasts from familial and sporadic forms of AD patients, due to the rise in the conversion of free cholesterol to cholesteryl esters and to phospholipid synthesis [162]. Imbalances in cholesterol homeostasis have been observed in AD and are believed to play a role in the progression of the disease by facilitating the accumulation of Aβ. Notably, increased mitochondrial cholesterol levels have been found to heighten susceptibility to neurotoxicity caused by Aβ. High levels of STARD3 at all ages analyzed were reported in APP/PS1 mice compared to wild-type mice, in which the levels of this protein were maintained steadily during aging [163]. Increased STARD3 levels may indicate an increase in mitochondrial lysosome contact mediated by this carrier for the cholesterol transfer (Figure 3), contributing to elevated mitochondrial cholesterol levels. Indeed, this accumulation was linked to elevated levels of proteins that facilitate the translocation of cholesterol to the mitochondria, starting from the ER [163]. In this context, the cholesterol transfer from the lysosome mediated by high levels of STARD3 may be involved in the accumulation process contributing to the AD progression in old APP/PS1 transgenic mice. Furthermore, as already discussed, three-way contacts are common for organelle communication. In particular, lysosomes directly share contact sites with the ER and mitochondria, possibly forming a tripartite complex [164]. Lysosomal dysfunction may be due to pH acidification or Ca2+ metabolism, which could be regulated and maintained through organelle contact sites [165].

In Table 2, alterations in mitochondria–lysosome tethering proteins levels and function and the investigated models reported in the literature in the context of AD, were summarized.

The concept that lysosomal dysfunction could affect communication between organelles via contact sites, such as those located at the endoplasmic reticulum and mitochondria, is intriguing. The alteration of MCSs may ultimately compromise the entire neuronal network, resulting in neuron degeneration. Therefore, the link between intracellular dysfunctions and the onset of AD may originate from abnormalities in organelles, which can interfere with their communication and impact cellular homeostasis.

## 7. Conclusions

Although the exact role of mitochondria–lysosome MCSs in AD remains unclear, the cellular processes they regulate in healthy cells are reported to be disrupted in neurons of patients with AD. Indeed, alterations in mitochondrial fission, as well as in the exchange of essential ions like calcium and iron, which are fundamental for many cellular processes, of lipids, and other molecules, have been observed in AD, playing a significant role in the loss of cellular homeostasis leading to neural impairment. Therefore, mitochondria–lysosome MCSs may have a fundamental role in AD pathogenesis, and their alteration may contribute to neuronal degeneration. More advanced and easy-to-use techniques are needed to gain a deeper understanding of the regulation and functions of MCSs, which could provide a new perspective into the disease and open promising avenues and new targets for AD therapies.

## Figures and Tables

**Table 1:** Benefits and disadvantages of the techniques reported in the literature for studying mitochondria–lysosome MCSs.

Methods	Benefits	Disadvantages	References
Electron Microscopy-based techniques			
EM	High-resolution imaging and magnification	Requires chemical fixation that may induce artifacts Inability to image live cells Creation of monochromatic images	[16]
TEM	High-resolution ultrastructure imaging Provides information on both the structure and elemental composition	Low-throughput technique Potential for artifacts Inability to image live cells	[46,67]
FIB-SEM	3D reconstruction through sequential imaging for detailed structural analysis Allows the study of contact dynamics High resolution	Slow data acquisition High-maintenance equipment Technically demanding	[69]
ET	Visualization of the full, native 3D architecture of complex samples like organelles Artifacts minimization	Limited tilting range of sample holder Technically demanding Inability to observe live cells	[67]
Super Resolution Fluorescent Microscopy-based techniques			
SIM	Live cell imaging and dynamics Fast image acquisition speed Low phototoxicity 3D structure imaging Compatibility with conventional fluorescent probes	Lower lateral resolution Creation of artifacts during image reconstruction	[16,73,77]
STORM	Live-cell imaging and time-lapse High spatial resolution (10–30 nm) Individual multicolor fluorophore localization Compatibility with conventional fluorescent probes and organic dyes	Slow image acquisition Potential for photobleaching and phototoxicity	[52]
CLEM	Combination of fluorescence microscopy (multicolor labels) and electron microscopy (high resolution) Localization of molecules with the corresponding ultrastructural context	Low efficiency of available combinatorial probes Different requirements for sample preparation for the two techniques	[16,69,70]
Proximity-based biotinylation and fluorescence techniques			
APEX2	Rapid reaction and high spatiotemporal resolution High activity and specificity	High oxidative stress and toxicity due to H_2_O_2_	[70]
FRET	Measures molecular distances in living cells in real-time High spatial resolution and sensitivity Can be used to study the dynamics of MCS	Requires the same expression of the donor and acceptor Possible photobleaching, cross-excitation, autofluorescence from cellular components, and bleed-through emission	[16]
SPLICS	*In vitro* and *in vivo* applications	Irreversibility of the GFP reconstitution	[34]

Abbreviation: EM Electron Microscopy; TEM Transmission Electron Microscopy; FIB-SEM Focused Ion Beam-Scanning Electron Microscope; ET electron tomography; SIM Structured Illumination Microscopy; STORM STochastic Optical Reconstruction Microscopy; CLEM Correlative Light and Electron Microscopy; FRET Fluorescence Resonance Energy Transfer.

**Table 2:** Alterations of tethering protein levels in the context of AD.

Tethering Proteins at MCS		Protein Alteration in AD	Evidence for Dysfunction	Investigated Models	Reference
RAB7A-TBC1D15-FIS1	FIS1	Increased levels	Abnormal mitochondrial dynamics (fission)	AD brains and APP transgenic mice primary hippocampal neurons	[126,127]
	RAB7A	Increased levels	Excessive mitochondrial fission Regulation of tau secretion	Neurons and CSF of MCI and AD patients	[129,130,131,132,133]
	TBC1D15	No information			
Mfn2		Reduced levels	Abnormal mitochondrial fusion and fragmentation Impaired iron transfer	AD brains and primary hippocampal neurons from APP transgenic mice, AD cybrids	[126,127,134]
LAMP1-GDAP1	LAMP1	Increased levels	Impaired lysosomal functions Accumulation of lysosomes in amyloid plaques	CSF, neurons, glial cells of AD patients; AD mouse models,	[132,146,147,148]
	LAMP1	Reduced levels	Lysosomal dysfunction	AD mouse and cell models	[149]
	GDAP1	No information			
VDAC1-TRPML1	VDAC1	Increased levels	Impaired calcium signaling Mitochondrial dysfunction	AD brains and cortical tissues from AD mouse models	[151,152]
	TRPML1	Reduced levels	Impaired calcium signaling Autophagy dysregulation	AD mouse models	[159,160]
STAD3		Increased levels	Increased mitochondrial cholesterol levels	AD mouse models	[163]

Abbreviations: MCI Mild Cognitive Impairment; CSF Cerebrospinal Fluid.

## Data Availability

Not applicable.

## References

[1] Safiri S., Ghaffari Jolfayi A., Fazlollahi A., Morsali S., Sarkesh A., Daei Sorkhabi A., Golabi B., Aletaha R., Motlagh Asghari K., Hamidi S. (2024). Alzheimer’s Disease: A Comprehensive Review of Epidemiology, Risk Factors, Symptoms Diagnosis, Management, Caregiving, Advanced Treatments and Associated Challenges. Front. Med..

[2] Wang Y., Yang J. (2024). ER-Organelle Contacts: A Signaling Hub for Neurological Diseases. Pharmacol. Res..

[3] Voeltz G.K., Sawyer E.M., Hajnóczky G., Prinz W.A. (2024). Making the Connection: How Membrane Contact Sites Have Changed Our View of Organelle Biology. Cell.

[4] Bohnert M. (2020). Tether Me, Tether Me Not-Dynamic Organelle Contact Sites in Metabolic Rewiring. Dev. Cell.

[5] Reinisch K.M., De Camilli P., Melia T.J. (2025). Lipid Dynamics at Membrane Contact Sites. Annu. Rev. Biochem..

[6] Rakotonirina-Ricquebourg R., Costa V., Teixeira V. (2022). Hello from the Other Side: Membrane Contact of Lipid Droplets with Other Organelles and Subsequent Functional Implications. Prog. Lipid Res..

[7] Kozjak-Pavlovic V. (2017). The MICOS Complex of Human Mitochondria. Cell Tissue Res..

[8] Eramo M.J., Lisnyak V., Formosa L.E., Ryan M.T. (2020). The “mitochondrial Contact Site and Cristae Organising System” (MICOS) in Health and Human Disease. J. Biochem..

[9] Rizzuto R., Pinton P., Carrington W., Fay F.S., Fogarty K.E., Lifshitz L.M., Tuft R.A., Pozzan T. (1998). Close Contacts with the Endoplasmic Reticulum as Determinants of Mitochondrial Ca^2+^ Responses. Science.

[10] Bernhard W., Rouiller C. (1956). Close Topographical Relationship between Mitochondria and Ergastoplasm of Liver Cells in a Definite Phase of Cellular Activity. J. Biophys. Biochem. Cytol..

[11] Calì T., Bayer E.M., Eden E.R., Hajnóczky G., Kornmann B., Lackner L., Liou J., Reinisch K., Rhee H.-W., Rizzuto R. (2025). Key Challenges and Recommendations for Defining Organelle Membrane Contact Sites. Nat. Rev. Mol. Cell Biol..

[12] Giacomello M., Pyakurel A., Glytsou C., Scorrano L. (2020). The Cell Biology of Mitochondrial Membrane Dynamics. Nat. Rev. Mol. Cell Biol..

[13] Petkovic M., O’Brien C.E., Jan Y.N. (2021). Interorganelle Communication, Aging, and Neurodegeneration. Genes Dev..

[14] Prinz W.A., Toulmay A., Balla T. (2020). The Functional Universe of Membrane Contact Sites. Nat. Rev. Mol. Cell Biol..

[15] Mc Donald J.M., Krainc D. (2017). Lysosomal Proteins as a Therapeutic Target in Neurodegeneration. Annu. Rev. Med..

[16] Wong Y.C., Ysselstein D., Krainc D. (2018). Mitochondria–Lysosome Contacts Regulate Mitochondrial Fission via RAB7 GTP Hydrolysis. Nature.

[17] Deus C.M., Yambire K.F., Oliveira P.J., Raimundo N. (2020). Mitochondria-Lysosome Crosstalk: From Physiology to Neurodegeneration. Trends Mol. Med..

[18] Cisneros J., Belton T.B., Shum G.C., Molakal C.G., Wong Y.C. (2022). Mitochondria-Lysosome Contact Site Dynamics and Misregulation in Neurodegenerative Diseases. Trends Neurosci..

[19] Cioni J.-M., Lin J.Q., Holtermann A.V., Koppers M., Jakobs M.A.H., Azizi A., Turner-Bridger B., Shigeoka T., Franze K., Harris W.A. (2019). Late Endosomes Act as mRNA Translation Platforms and Sustain Mitochondria in Axons. Cell.

[20] Cantarero L., Juárez-Escoto E., Civera-Tregón A., Rodríguez-Sanz M., Roldán M., Benítez R., Hoenicka J., Palau F. (2021). Mitochondria-Lysosome Membrane Contacts Are Defective in GDAP1-Related Charcot-Marie-Tooth Disease. Hum. Mol. Genet..

[21] Wong Y.C., Peng W., Krainc D. (2019). Lysosomal Regulation of Inter-Mitochondrial Contact Fate and Motility in Charcot-Marie-Tooth Type 2. Dev. Cell.

[22] Burbulla L.F., Jeon S., Zheng J., Song P., Silverman R.B., Krainc D. (2019). A Modulator of Wild-Type Glucocerebrosidase Improves Pathogenic Phenotypes in Dopaminergic Neuronal Models of Parkinson’s Disease. Sci. Transl. Med..

[23] Rabas N., Palmer S., Mitchell L., Ismail S., Gohlke A., Riley J.S., Tait S.W.G., Gammage P., Soares L.L., Macpherson I.R. (2021). PINK1 Drives Production of mtDNA-Containing Extracellular Vesicles to Promote Invasiveness. J. Cell Biol..

[24] Kim S., Wong Y.C., Gao F., Krainc D. (2021). Dysregulation of Mitochondria-Lysosome Contacts by GBA1 Dysfunction in Dopaminergic Neuronal Models of Parkinson’s Disease. Nat. Commun..

[25] Höglinger D., Burgoyne T., Sanchez-Heras E., Hartwig P., Colaco A., Newton J., Futter C.E., Spiegel S., Platt F.M., Eden E.R. (2019). NPC1 Regulates ER Contacts with Endocytic Organelles to Mediate Cholesterol Egress. Nat. Commun..

[26] Peng W., Wong Y.C., Krainc D. (2020). Mitochondria-Lysosome Contacts Regulate Mitochondrial Ca^2+^ Dynamics via Lysosomal TRPML1. Proc. Natl. Acad. Sci. USA.

[27] Suomalainen A., Nunnari J. (2024). Mitochondria at the Crossroads of Health and Disease. Cell.

[28] Rizzollo F., More S., Vangheluwe P., Agostinis P. (2021). The Lysosome as a Master Regulator of Iron Metabolism. Trends Biochem. Sci..

[29] Settembre C., Perera R.M. (2024). Lysosomes as Coordinators of Cellular Catabolism, Metabolic Signalling and Organ Physiology. Nat. Rev. Mol. Cell Biol..

[30] Sugiura A., McLelland G.-L., Fon E.A., McBride H.M. (2014). A New Pathway for Mitochondrial Quality Control: Mitochondrial-Derived Vesicles. EMBO J..

[31] Wong Y.C., Kim S., Peng W., Krainc D. (2019). Regulation and Function of Mitochondria-Lysosome Membrane Contact Sites in Cellular Homeostasis. Trends Cell Biol..

[32] Boutry M., Kim P.K. (2021). ORP1L Mediated PI(4)P Signaling at ER-Lysosome-Mitochondrion Three-Way Contact Contributes to Mitochondrial Division. Nat. Commun..

[33] Juhl A.D., Heegaard C.W., Werner S., Schneider G., Krishnan K., Covey D.F., Wüstner D. (2021). Quantitative Imaging of Membrane Contact Sites for Sterol Transfer between Endo-Lysosomes and Mitochondria in Living Cells. Sci. Rep..

[34] Giamogante F., Barazzuol L., Maiorca F., Poggio E., Esposito A., Masato A., Napolitano G., Vagnoni A., Calì T., Brini M. (2024). A SPLICS Reporter Reveals α-Synuclein Regulation of Lysosome-Mitochondria Contacts Which Affects TFEB Nuclear Translocation. Nat. Commun..

[35] Peng W., Schröder L.F., Song P., Wong Y.C., Krainc D. (2023). Parkin Regulates Amino Acid Homeostasis at Mitochondria-Lysosome (M/L) Contact Sites in Parkinson’s Disease. Sci. Adv..

[36] Guerra F., Bucci C. (2016). Multiple Roles of the Small GTPase Rab7. Cells.

[37] Bucci C., Thomsen P., Nicoziani P., McCarthy J., van Deurs B. (2000). Rab7: A Key to Lysosome Biogenesis. Mol. Biol. Cell.

[38] Yu W., Sun S., Xu H., Li C., Ren J., Zhang Y. (2020). TBC1D15/RAB7-Regulated Mitochondria-Lysosome Interaction Confers Cardioprotection against Acute Myocardial Infarction-Induced Cardiac Injury. Theranostics.

[39] Onoue K., Jofuku A., Ban-Ishihara R., Ishihara T., Maeda M., Koshiba T., Itoh T., Fukuda M., Otera H., Oka T. (2013). Fis1 Acts as a Mitochondrial Recruitment Factor for TBC1D15 That Is Involved in Regulation of Mitochondrial Morphology. J. Cell Sci..

[40] Zhang X.-M., Walsh B., Mitchell C.A., Rowe T. (2005). TBC Domain Family, Member 15 Is a Novel Mammalian Rab GTPase-Activating Protein with Substrate Preference for Rab7. Biochem. Biophys. Res. Commun..

[41] Jumper J., Evans R., Pritzel A., Green T., Figurnov M., Ronneberger O., Tunyasuvunakool K., Bates R., Žídek A., Potapenko A. (2021). Highly Accurate Protein Structure Prediction with AlphaFold. Nature.

[42] Varadi M., Bertoni D., Magana P., Paramval U., Pidruchna I., Radhakrishnan M., Tsenkov M., Nair S., Mirdita M., Yeo J. (2024). AlphaFold Protein Structure Database in 2024: Providing Structure Coverage for over 214 Million Protein Sequences. Nucleic Acids Res..

[43] Muñoz-Braceras S., Tornero-Écija A.R., Vincent O., Escalante R. (2019). VPS13A Is Closely Associated with Mitochondria and Is Required for Efficient Lysosomal Degradation. Dis. Model. Mech..

[44] Kleele T., Rey T., Winter J., Zaganelli S., Mahecic D., Perreten Lambert H., Ruberto F.P., Nemir M., Wai T., Pedrazzini T. (2021). Distinct Fission Signatures Predict Mitochondrial Degradation or Biogenesis. Nature.

[45] Langemeyer L., Fröhlich F., Ungermann C. (2018). Rab GTPase Function in Endosome and Lysosome Biogenesis. Trends Cell Biol..

[46] Khalil S., Holy M., Grado S., Fleming R., Kurita R., Nakamura Y., Goldfarb A. (2017). A Specialized Pathway for Erythroid Iron Delivery through Lysosomal Trafficking of Transferrin Receptor 2. Blood Adv..

[47] Wang N., Wang X., Lan B., Gao Y., Cai Y. (2025). DRP1, Fission and Apoptosis. Cell Death Discov..

[48] Siow W.X., Kabiri Y., Tang R., Chao Y.-K., Plesch E., Eberhagen C., Flenkenthaler F., Fröhlich T., Bracher F., Grimm C. (2022). Lysosomal TRPML1 Regulates Mitochondrial Function in Hepatocellular Carcinoma Cells. J. Cell Sci..

[49] Baughman J.M., Perocchi F., Girgis H.S., Plovanich M., Belcher-Timme C.A., Sancak Y., Bao X.R., Strittmatter L., Goldberger O., Bogorad R.L. (2011). Integrative Genomics Identifies MCU as an Essential Component of the Mitochondrial Calcium Uniporter. Nature.

[50] De Stefani D., Raffaello A., Teardo E., Szabò I., Rizzuto R. (2011). A Forty-Kilodalton Protein of the Inner Membrane Is the Mitochondrial Calcium Uniporter. Nature.

[51] Hamdi A., Roshan T.M., Kahawita T.M., Mason A.B., Sheftel A.D., Ponka P. (2016). Erythroid Cell Mitochondria Receive Endosomal Iron by a “Kiss-and-Run” Mechanism. Biochim. Biophys. Acta.

[52] Das A., Nag S., Mason A.B., Barroso M.M. (2016). Endosome-Mitochondria Interactions Are Modulated by Iron Release from Transferrin. J. Cell Biol..

[53] Rizzollo F., Agostinis P. (2025). Mitochondria-Lysosome Contact Sites: Emerging Players in Cellular Homeostasis and Disease. Contact.

[54] Bao L., Liu Q., Wang J., Shi L., Pang Y., Niu Y., Zhang R. (2024). The Interactions of Subcellular Organelles in Pulmonary Fibrosis Induced by Carbon Black Nanoparticles: A Comprehensive Review. Arch. Toxicol..

[55] Zhao T., Huang X., Han L., Wang X., Cheng H., Zhao Y., Chen Q., Chen J., Cheng H., Xiao R. (2012). Central Role of Mitofusin 2 in Autophagosome-Lysosome Fusion in Cardiomyocytes. J. Biol. Chem..

[56] Filadi R., Pendin D., Pizzo P. (2018). Mitofusin 2: From Functions to Disease. Cell Death Dis..

[57] Agostinis P., Rizzollo F., Escamilla-Ayala A., Fattorelli N., Lysiak N., More S., Barazzuol L., Haute C.V.D., Asselberghs J.V., Nittner D. (2024). A Bdh2-Driven Lysosome to Mitochondria Iron Trafficking Controls Ferroptosis in Melanoma. Research Square.

[58] Yambire K.F., Rostosky C., Watanabe T., Pacheu-Grau D., Torres-Odio S., Sanchez-Guerrero A., Senderovich O., Meyron-Holtz E.G., Milosevic I., Frahm J. (2019). Impaired Lysosomal Acidification Triggers Iron Deficiency and Inflammation in Vivo. eLife.

[59] Weber R.A., Yen F.S., Nicholson S.P.V., Alwaseem H., Bayraktar E.C., Alam M., Timson R.C., La K., Abu-Remaileh M., Molina H. (2020). Maintaining Iron Homeostasis Is the Key Role of Lysosomal Acidity for Cell Proliferation. Mol. Cell.

[60] Sassano M.L., Felipe-Abrio B., Agostinis P. (2022). ER-Mitochondria Contact Sites; a Multifaceted Factory for Ca^2+^ Signaling and Lipid Transport. Front. Cell Dev. Biol..

[61] Charman M., Kennedy B.E., Osborne N., Karten B. (2010). MLN64 Mediates Egress of Cholesterol from Endosomes to Mitochondria in the Absence of Functional Niemann-Pick Type C1 Protein. J. Lipid Res..

[62] Hönscher C., Mari M., Auffarth K., Bohnert M., Griffith J., Geerts W., van der Laan M., Cabrera M., Reggiori F., Ungermann C. (2014). Cellular Metabolism Regulates Contact Sites between Vacuoles and Mitochondria. Dev. Cell.

[63] González Montoro A., Auffarth K., Hönscher C., Bohnert M., Becker T., Warscheid B., Reggiori F., van der Laan M., Fröhlich F., Ungermann C. (2018). Vps39 Interacts with Tom40 to Establish One of Two Functionally Distinct Vacuole-Mitochondria Contact Sites. Dev. Cell.

[64] Bean B.D.M., Dziurdzik S.K., Kolehmainen K.L., Fowler C.M.S., Kwong W.K., Grad L.I., Davey M., Schluter C., Conibear E. (2018). Competitive Organelle-Specific Adaptors Recruit Vps13 to Membrane Contact Sites. J. Cell Biol..

[65] Pickles S., Vigié P., Youle R.J. (2018). Mitophagy and Quality Control Mechanisms in Mitochondrial Maintenance. Curr. Biol..

[66] Scorrano L., De Matteis M.A., Emr S., Giordano F., Hajnóczky G., Kornmann B., Lackner L.L., Levine T.P., Pellegrini L., Reinisch K. (2019). Coming Together to Define Membrane Contact Sites. Nat. Commun..

[67] Aston D., Capel R.A., Ford K.L., Christian H.C., Mirams G.R., Rog-Zielinska E.A., Kohl P., Galione A., Burton R.A.B., Terrar D.A. (2017). High Resolution Structural Evidence Suggests the Sarcoplasmic Reticulum Forms Microdomains with Acidic Stores (Lysosomes) in the Heart. Sci. Rep..

[68] Martell J.D., Deerinck T.J., Lam S.S., Ellisman M.H., Ting A.Y. (2017). Electron Microscopy Using the Genetically Encoded APEX2 Tag in Cultured Mammalian Cells. Nat. Protoc..

[69] Fermie J., Liv N., Ten Brink C., van Donselaar E.G., Müller W.H., Schieber N.L., Schwab Y., Gerritsen H.C., Klumperman J. (2018). Single Organelle Dynamics Linked to 3D Structure by Correlative Live-Cell Imaging and 3D Electron Microscopy. Traffic.

[70] Jung M., Mun J.Y. (2024). Dual-Color Correlative Light and Electron Microscopy for the Visualization of Interactions between Mitochondria and Lysosomes. J. Vis. Exp..

[71] Saibil H.R. (2022). Cryo-EM in Molecular and Cellular Biology. Mol. Cell.

[72] Ching C., Maufront J., di Cicco A., Lévy D., Dezi M. (2024). Cool-Contacts: Cryo-Electron Microscopy of Membrane Contact Sites and Their Components. Contact.

[73] Han Y., Li M., Qiu F., Zhang M., Zhang Y.-H. (2017). Cell-Permeable Organic Fluorescent Probes for Live-Cell Long-Term Super-Resolution Imaging Reveal Lysosome-Mitochondrion Interactions. Nat. Commun..

[74] Nieto-Garai J.A., Olazar-Intxausti J., Anso I., Lorizate M., Terrones O., Contreras F.-X. (2022). Super-Resolution Microscopy to Study Interorganelle Contact Sites. Int. J. Mol. Sci..

[75] Lu M., Ward E., van Tartwijk F.W., Kaminski C.F. (2021). Advances in the Study of Organelle Interactions and Their Role in Neurodegenerative Diseases Enabled by Super-Resolution Microscopy. Neurobiol. Dis..

[76] Cieri D., Vicario M., Giacomello M., Vallese F., Filadi R., Wagner T., Pozzan T., Pizzo P., Scorrano L., Brini M. (2018). SPLICS: A Split Green Fluorescent Protein-Based Contact Site Sensor for Narrow and Wide Heterotypic Organelle Juxtaposition. Cell Death Differ..

[77] Chen Q., Jin C., Shao X., Guan R., Tian Z., Wang C., Liu F., Ling P., Guan J.-L., Ji L. (2018). Super-Resolution Tracking of Mitochondrial Dynamics with An Iridium(III) Luminophore. Small.

[78] Rostagno A.A. (2022). Pathogenesis of Alzheimer’s Disease. Int. J. Mol. Sci..

[79] Lapierre L.R., Kumsta C., Sandri M., Ballabio A., Hansen M. (2015). Transcriptional and Epigenetic Regulation of Autophagy in Aging. Autophagy.

[80] Cannizzo E.S., Clement C.C., Morozova K., Valdor R., Kaushik S., Almeida L.N., Follo C., Sahu R., Cuervo A.M., Macian F. (2012). Age-Related Oxidative Stress Compromises Endosomal Proteostasis. Cell Rep..

[81] Chou C.-C., Vest R., Prado M.A., Wilson-Grady J., Paulo J.A., Shibuya Y., Moran-Losada P., Lee T.-T., Luo J., Gygi S.P. (2025). Proteostasis and Lysosomal Repair Deficits in Transdifferentiated Neurons of Alzheimer’s Disease. Nat. Cell Biol..

[82] Kaushik S., Cuervo A.M. (2015). Proteostasis and Aging. Nat. Med..

[83] Chen H.-K., Ji Z.-S., Dodson S.E., Miranda R.D., Rosenblum C.I., Reynolds I.J., Freedman S.B., Weisgraber K.H., Huang Y., Mahley R.W. (2011). Apolipoprotein E4 Domain Interaction Mediates Detrimental Effects on Mitochondria and Is a Potential Therapeutic Target for Alzheimer Disease. J. Biol. Chem..

[84] Simonovitch S., Schmukler E., Masliah E., Pinkas-Kramarski R., Michaelson D.M. (2019). The Effects of APOE4 on Mitochondrial Dynamics and Proteins in Vivo. J. Alzheimers Dis..

[85] Schmukler E., Solomon S., Simonovitch S., Goldshmit Y., Wolfson E., Michaelson D.M., Pinkas-Kramarski R. (2020). Altered Mitochondrial Dynamics and Function in APOE4-Expressing Astrocytes. Cell Death Dis..

[86] Lin M.T., Beal M.F. (2006). Mitochondrial Dysfunction and Oxidative Stress in Neurodegenerative Diseases. Nature.

[87] Wallace D.C. (2005). A Mitochondrial Paradigm of Metabolic and Degenerative Diseases, Aging, and Cancer: A Dawn for Evolutionary Medicine. Annu. Rev. Genet..

[88] Wei W., Keogh M.J., Wilson I., Coxhead J., Ryan S., Rollinson S., Griffin H., Kurzawa-Akanbi M., Santibanez-Koref M., Talbot K. (2017). Mitochondrial DNA Point Mutations and Relative Copy Number in 1363 Disease and Control Human Brains. Acta Neuropathol. Commun..

[89] Coskun P.E., Beal M.F., Wallace D.C. (2004). Alzheimer’s Brains Harbor Somatic mtDNA Control-Region Mutations That Suppress Mitochondrial Transcription and Replication. Proc. Natl. Acad. Sci. USA.

[90] Reiss A.B., Gulkarov S., Jacob B., Srivastava A., Pinkhasov A., Gomolin I.H., Stecker M.M., Wisniewski T., De Leon J. (2024). Mitochondria in Alzheimer’s Disease Pathogenesis. Life.

[91] Reutzel M., Grewal R., Joppe A., Eckert G.P. (2022). Age-Dependent Alterations of Cognition, Mitochondrial Function, and Beta-Amyloid Deposition in a Murine Model of Alzheimer’s Disease-A Longitudinal Study. Front. Aging Neurosci..

[92] D’Alessandro M.C.B., Kanaan S., Geller M., Praticò D., Daher J.P.L. (2025). Mitochondrial Dysfunction in Alzheimer’s Disease. Ageing Res. Rev..

[93] Jayatunga D.P.W., Hone E., Bharadwaj P., Garg M., Verdile G., Guillemin G.J., Martins R.N. (2020). Targeting Mitophagy in Alzheimer’s Disease. J. Alzheimers Dis..

[94] Kazemeini S., Nadeem-Tariq A., Shih R., Rafanan J., Ghani N., Vida T.A. (2024). From Plaques to Pathways in Alzheimer’s Disease: The Mitochondrial-Neurovascular-Metabolic Hypothesis. Int. J. Mol. Sci..

[95] Swerdlow R.H., Burns J.M., Khan S.M. (2014). The Alzheimer’s Disease Mitochondrial Cascade Hypothesis: Progress and Perspectives. Biochim. Biophys. Acta.

[96] Kametani F., Hasegawa M. (2018). Reconsideration of Amyloid Hypothesis and Tau Hypothesis in Alzheimer’s Disease. Front. Neurosci..

[97] Ashleigh T., Swerdlow R.H., Beal M.F. (2023). The Role of Mitochondrial Dysfunction in Alzheimer’s Disease Pathogenesis. Alzheimers Dement..

[98] Spina E., Ferrari R.R., Pellegrini E., Colombo M., Poloni T.E., Guaita A., Davin A. (2025). Mitochondrial Alterations, Oxidative Stress, and Therapeutic Implications in Alzheimer’s Disease: A Narrative Review. Cells.

[99] McGill Percy K.C., Liu Z., Qi X. (2025). Mitochondrial Dysfunction in Alzheimer’s Disease: Guiding the Path to Targeted Therapies. Neurotherapeutics.

[100] Colacurcio D.J., Nixon R.A. (2016). Disorders of Lysosomal Acidification-The Emerging Role of v-ATPase in Aging and Neurodegenerative Disease. Ageing Res. Rev..

[101] Menzies F.M., Fleming A., Rubinsztein D.C. (2015). Compromised Autophagy and Neurodegenerative Diseases. Nat. Rev. Neurosci..

[102] Orr M.E., Oddo S. (2013). Autophagic/Lysosomal Dysfunction in Alzheimer’s Disease. Alzheimers Res. Ther..

[103] Heo H., Park H., Lee M.S., Kim J., Kim J., Jung S.-Y., Kim S.K., Lee S., Chang J. (2024). TRIM22 Facilitates Autophagosome-Lysosome Fusion by Mediating the Association of GABARAPs and PLEKHM1. Autophagy.

[104] Nixon R.A., Wegiel J., Kumar A., Yu W.H., Peterhoff C., Cataldo A., Cuervo A.M. (2005). Extensive Involvement of Autophagy in Alzheimer Disease: An Immuno-Electron Microscopy Study. J. Neuropathol. Exp. Neurol..

[105] Lo C.H., Zeng J. (2023). Defective Lysosomal Acidification: A New Prognostic Marker and Therapeutic Target for Neurodegenerative Diseases. Transl. Neurodegener..

[106] Lee J.-H., Yang D.-S., Goulbourne C.N., Im E., Stavrides P., Pensalfini A., Chan H., Bouchet-Marquis C., Bleiwas C., Berg M.J. (2022). Faulty Autolysosome Acidification in Alzheimer’s Disease Mouse Models Induces Autophagic Build-up of Aβ in Neurons, Yielding Senile Plaques. Nat. Neurosci..

[107] Cataldo A.M., Nixon R.A. (1990). Enzymatically Active Lysosomal Proteases Are Associated with Amyloid Deposits in Alzheimer Brain. Proc. Natl. Acad. Sci. USA.

[108] Nixon R.A. (2007). Autophagy, Amyloidogenesis and Alzheimer Disease. J. Cell Sci..

[109] Aman Y., Schmauck-Medina T., Hansen M., Morimoto R.I., Simon A.K., Bjedov I., Palikaras K., Simonsen A., Johansen T., Tavernarakis N. (2021). Autophagy in Healthy Aging and Disease. Nat. Aging.

[110] Van Acker Z.P., Bretou M., Annaert W. (2019). Endo-Lysosomal Dysregulations and Late-Onset Alzheimer’s Disease: Impact of Genetic Risk Factors. Mol. Neurodegener..

[111] Ditaranto K., Tekirian T.L., Yang A.J. (2001). Lysosomal Membrane Damage in Soluble Abeta-Mediated Cell Death in Alzheimer’s Disease. Neurobiol. Dis..

[112] Kosenko E., Poghosyan A., Kaminsky Y. (2011). Subcellular Compartmentalization of Proteolytic Enzymes in Brain Regions and the Effects of Chronic β-Amyloid Treatment. Brain Res..

[113] Zaretsky D.V., Zaretskaia M.V., Molkov Y.I. (2022). Membrane Channel Hypothesis of Lysosomal Permeabilization by Beta-Amyloid. Neurosci. Lett..

[114] Sanyal A., Scanavachi G., Somerville E., Saminathan A., Nair A., Bango Da Cunha Correia R.F., Aylan B., Sitarska E., Oikonomou A., Hatzakis N.S. (2025). Neuronal Constitutive Endolysosomal Perforations Enable α-Synuclein Aggregation by Internalized PFFs. J. Cell Biol..

[115] Rose K., Jepson T., Shukla S., Maya-Romero A., Kampmann M., Xu K., Hurley J.H. (2024). Tau Fibrils Induce Nanoscale Membrane Damage and Nucleate Cytosolic Tau at Lysosomes. Proc. Natl. Acad. Sci. USA.

[116] Kim Y., Ha T.-Y., Lee M.-S., Chang K.-A. (2025). Regulatory Mechanisms and Therapeutic Implications of Lysosomal Dysfunction in Alzheimer’s Disease. Int. J. Biol. Sci..

[117] Angst G., Jia N., Esqueda L.E.T., Fan Y., Cai Q., Wang C. (2025). Autophagy in Alzheimer Disease Pathogenesis and Its Therapeutic Values. Autophagy Rep..

[118] Demers-Lamarche J., Guillebaud G., Tlili M., Todkar K., Bélanger N., Grondin M., Nguyen A.P., Michel J., Germain M. (2016). Loss of Mitochondrial Function Impairs Lysosomes. J. Biol. Chem..

[119] Baixauli F., Acín-Pérez R., Villarroya-Beltrí C., Mazzeo C., Nuñez-Andrade N., Gabandé-Rodriguez E., Ledesma M.D., Blázquez A., Martin M.A., Falcón-Pérez J.M. (2015). Mitochondrial Respiration Controls Lysosomal Function during Inflammatory T Cell Responses. Cell Metab..

[120] Fernandez-Mosquera L., Yambire K.F., Couto R., Pereyra L., Pabis K., Ponsford A.H., Diogo C.V., Stagi M., Milosevic I., Raimundo N. (2019). Mitochondrial Respiratory Chain Deficiency Inhibits Lysosomal Hydrolysis. Autophagy.

[121] Joshi A.U., Ebert A.E., Haileselassie B., Mochly-Rosen D. (2019). Drp1/Fis1-Mediated Mitochondrial Fragmentation Leads to Lysosomal Dysfunction in Cardiac Models of Huntington’s Disease. J. Mol. Cell Cardiol..

[122] Girolimetti G., Gagliardi S., Cordella P., Bramato G., Di Corato R., Romano R., Guerra F., Bucci C. (2025). Induced Mitochondrial Deficit by NDUFS3 Transient Silencing Reduces RAB7 Expression and Causes Lysosomal Dysfunction in Pancreatic Cancer Cells. Cell Commun. Signal..

[123] Hughes A.L., Gottschling D.E. (2012). An Early Age Increase in Vacuolar pH Limits Mitochondrial Function and Lifespan in Yeast. Nature.

[124] Jiang Y., Sato Y., Im E., Berg M., Bordi M., Darji S., Kumar A., Mohan P.S., Bandyopadhyay U., Diaz A. (2019). Lysosomal Dysfunction in Down Syndrome Is APP-Dependent and Mediated by APP-βCTF (C99). J. Neurosci..

[125] Bonda D.J., Wang X., Perry G., Smith M.A., Zhu X. (2010). Mitochondrial Dynamics in Alzheimer’s Disease: Opportunities for Future Treatment Strategies. Drugs Aging.

[126] Lian W.-W., Zhou W., Zhang B.-Y., Jia H., Xu L.-J., Liu A.-L., Du G.-H. (2021). DL0410 Ameliorates Cognitive Disorder in SAMP8 Mice by Promoting Mitochondrial Dynamics and the NMDAR-CREB-BDNF Pathway. Acta Pharmacol. Sin..

[127] Manczak M., Calkins M.J., Reddy P.H. (2011). Impaired Mitochondrial Dynamics and Abnormal Interaction of Amyloid Beta with Mitochondrial Protein Drp1 in Neurons from Patients with Alzheimer’s Disease: Implications for Neuronal Damage. Hum. Mol. Genet..

[128] Ginsberg S.D., Mufson E.J., Counts S.E., Wuu J., Alldred M.J., Nixon R.A., Che S. (2010). Regional Selectivity of Rab5 and Rab7 Protein Upregulation in Mild Cognitive Impairment and Alzheimer’s Disease. J. Alzheimers Dis..

[129] Ginsberg S.D., Mufson E.J., Alldred M.J., Counts S.E., Wuu J., Nixon R.A., Che S. (2011). Upregulation of Select Rab GTPases in Cholinergic Basal Forebrain Neurons in Mild Cognitive Impairment and Alzheimer’s Disease. J. Chem. Neuroanat..

[130] Ginsberg S.D., Alldred M.J., Counts S.E., Cataldo A.M., Neve R.L., Jiang Y., Wuu J., Chao M.V., Mufson E.J., Nixon R.A. (2010). Microarray Analysis of Hippocampal CA1 Neurons Implicates Early Endosomal Dysfunction during Alzheimer’s Disease Progression. Biol. Psychiatry.

[131] Tiernan C.T., Ginsberg S.D., Guillozet-Bongaarts A.L., Ward S.M., He B., Kanaan N.M., Mufson E.J., Binder L.I., Counts S.E. (2016). Protein Homeostasis Gene Dysregulation in Pretangle-Bearing Nucleus Basalis Neurons during the Progression of Alzheimer’s Disease. Neurobiol. Aging.

[132] Armstrong A., Mattsson N., Appelqvist H., Janefjord C., Sandin L., Agholme L., Olsson B., Svensson S., Blennow K., Zetterberg H. (2014). Lysosomal Network Proteins as Potential Novel CSF Biomarkers for Alzheimer’s Disease. Neuromolecular Med..

[133] Rodriguez L., Mohamed N.-V., Desjardins A., Lippé R., Fon E.A., Leclerc N. (2017). Rab7A Regulates Tau Secretion. J. Neurochem..

[134] Du F., Yu Q., Yan S.S. (2021). PINK1 Activation Attenuates Impaired Neuronal-Like Differentiation and Synaptogenesis and Mitochondrial Dysfunction in Alzheimer’s Disease Trans-Mitochondrial Cybrid Cells. J. Alzheimers Dis..

[135] Han S., Nandy P., Austria Q., Siedlak S.L., Torres S., Fujioka H., Wang W., Zhu X. (2020). Mfn2 Ablation in the Adult Mouse Hippocampus and Cortex Causes Neuronal Death. Cells.

[136] Chen H., Xing H., Zhong C., Lin X., Chen R., Luo N., Chen L., Huang Y. (2024). METTL3 Confers Protection against Mitochondrial Dysfunction and Cognitive Impairment in an Alzheimer Disease Mouse Model by Upregulating Mfn2 via N6-Methyladenosine Modification. J. Neuropathol. Exp. Neurol..

[137] Kerr J.S., Adriaanse B.A., Greig N.H., Mattson M.P., Cader M.Z., Bohr V.A., Fang E.F. (2017). Mitophagy and Alzheimer’s Disease: Cellular and Molecular Mechanisms. Trends Neurosci..

[138] Caccamo A., Majumder S., Richardson A., Strong R., Oddo S. (2010). Molecular Interplay between Mammalian Target of Rapamycin (mTOR), Amyloid-Beta, and Tau: Effects on Cognitive Impairments. J. Biol. Chem..

[139] Rocchi A., Yamamoto S., Ting T., Fan Y., Sadleir K., Wang Y., Zhang W., Huang S., Levine B., Vassar R. (2017). A Becn1 Mutation Mediates Hyperactive Autophagic Sequestration of Amyloid Oligomers and Improved Cognition in Alzheimer’s Disease. PLoS Genet..

[140] Zhang Z., Yang X., Song Y.-Q., Tu J. (2021). Autophagy in Alzheimer’s Disease Pathogenesis: Therapeutic Potential and Future Perspectives. Ageing Res. Rev..

[141] Nilsson P., Loganathan K., Sekiguchi M., Matsuba Y., Hui K., Tsubuki S., Tanaka M., Iwata N., Saito T., Saido T.C. (2013). Aβ Secretion and Plaque Formation Depend on Autophagy. Cell Rep..

[142] Bordi M., Berg M.J., Mohan P.S., Peterhoff C.M., Alldred M.J., Che S., Ginsberg S.D., Nixon R.A. (2016). Autophagy Flux in CA1 Neurons of Alzheimer Hippocampus: Increased Induction Overburdens Failing Lysosomes to Propel Neuritic Dystrophy. Autophagy.

[143] Chung K.M., Hernández N., Sproul A.A., Yu W.H. (2019). Alzheimer’s Disease and the Autophagic-Lysosomal System. Neurosci. Lett..

[144] Lachance V., Wang Q., Sweet E., Choi I., Cai C.-Z., Zhuang X.-X., Zhang Y., Jiang J.L., Blitzer R.D., Bozdagi-Gunal O. (2019). Autophagy Protein NRBF2 Has Reduced Expression in Alzheimer’s Brains and Modulates Memory and Amyloid-Beta Homeostasis in Mice. Mol. Neurodegener..

[145] Pickford F., Masliah E., Britschgi M., Lucin K., Narasimhan R., Jaeger P.A., Small S., Spencer B., Rockenstein E., Levine B. (2008). The Autophagy-Related Protein Beclin 1 Shows Reduced Expression in Early Alzheimer Disease and Regulates Amyloid Beta Accumulation in Mice. J. Clin. Investig..

[146] Sharoar M.G., Palko S., Ge Y., Saido T.C., Yan R. (2021). Accumulation of Saposin in Dystrophic Neurites Is Linked to Impaired Lysosomal Functions in Alzheimer’s Disease Brains. Mol. Neurodegener..

[147] Barrachina M., Maes T., Buesa C., Ferrer I. (2006). Lysosome-Associated Membrane Protein 1 (LAMP-1) in Alzheimer’s Disease. Neuropathol. Appl. Neurobiol..

[148] Gowrishankar S., Yuan P., Wu Y., Schrag M., Paradise S., Grutzendler J., De Camilli P., Ferguson S.M. (2015). Massive Accumulation of Luminal Protease-Deficient Axonal Lysosomes at Alzheimer’s Disease Amyloid Plaques. Proc. Natl. Acad. Sci. USA.

[149] Zhou W., Xiao D., Zhao Y., Tan B., Long Z., Yu L., He G. (2021). Enhanced Autolysosomal Function Ameliorates the Inflammatory Response Mediated by the NLRP3 Inflammasome in Alzheimer’s Disease. Front. Aging Neurosci..

[150] León M., Prieto J., Molina-Navarro M.M., García-García F., Barneo-Muñoz M., Ponsoda X., Sáez R., Palau F., Dopazo J., Izpisua Belmonte J.C. (2023). Rapid Degeneration of iPSC-Derived Motor Neurons Lacking Gdap1 Engages a Mitochondrial-Sustained Innate Immune Response. Cell Death Discov..

[151] Shoshan-Barmatz V., Nahon-Crystal E., Shteinfer-Kuzmine A., Gupta R. (2018). VDAC1, Mitochondrial Dysfunction, and Alzheimer’s Disease. Pharmacol. Res..

[152] Kmita H., Messina A.A., De Pinto V. (2023). VDAC as a Cellular Hub: Docking Molecules and Interactions. Int. J. Mol. Sci..

[153] Pérez M.J., Ponce D.P., Aranguiz A., Behrens M.I., Quintanilla R.A. (2018). Mitochondrial Permeability Transition Pore Contributes to Mitochondrial Dysfunction in Fibroblasts of Patients with Sporadic Alzheimer’s Disease. Redox Biol..

[154] Song L., Tang Y., Law B.Y.K. (2024). Targeting Calcium Signaling in Alzheimer’s Disease: Challenges and Promising Therapeutic Avenues. Neural Regen. Res..

[155] Chaudhary B., Kumari S., Dhapola R., Sharma P., Paidlewar M., Vellingiri B., Medhi B., HariKrishnaReddy D. (2025). Calcium Dysregulation in Alzheimer’s Disease: Unraveling the Molecular Nexus of Neuronal Dysfunction and Therapeutic Opportunities. Biochem. Pharmacol..

[156] Li J., Yang D., Li Z., Zhao M., Wang D., Sun Z., Wen P., Dai Y., Gou F., Ji Y. (2023). PINK1/Parkin-Mediated Mitophagy in Neurodegenerative Diseases. Ageing Res. Rev..

[157] Mattson M.P. (2010). ER Calcium and Alzheimer’s Disease: In a State of Flux. Sci. Signal..

[158] Calvo-Rodriguez M., Hou S.S., Snyder A.C., Kharitonova E.K., Russ A.N., Das S., Fan Z., Muzikansky A., Garcia-Alloza M., Serrano-Pozo A. (2020). Increased Mitochondrial Calcium Levels Associated with Neuronal Death in a Mouse Model of Alzheimer’s Disease. Nat. Commun..

[159] Curcio-Morelli C., Charles F.A., Micsenyi M.C., Cao Y., Venugopal B., Browning M.F., Dobrenis K., Cotman S.L., Walkley S.U., Slaugenhaupt S.A. (2010). Macroautophagy Is Defective in Mucolipin-1-Deficient Mouse Neurons. Neurobiol. Dis..

[160] Zhang L., Fang Y., Cheng X., Lian Y., Xu H., Zeng Z., Zhu H. (2017). TRPML1 Participates in the Progression of Alzheimer’s Disease by Regulating the PPARγ/AMPK/Mtor Signalling Pathway. Cell Physiol. Biochem..

[161] Hou Y., Dan X., Babbar M., Wei Y., Hasselbalch S.G., Croteau D.L., Bohr V.A. (2019). Ageing as a Risk Factor for Neurodegenerative Disease. Nat. Rev. Neurol..

[162] Area-Gomez E., Del Carmen Lara Castillo M., Tambini M.D., Guardia-Laguarta C., de Groof A.J.C., Madra M., Ikenouchi J., Umeda M., Bird T.D., Sturley S.L. (2012). Upregulated Function of Mitochondria-Associated ER Membranes in Alzheimer Disease. EMBO J..

[163] Barbero-Camps E., Fernández A., Baulies A., Martinez L., Fernández-Checa J.C., Colell A. (2014). Endoplasmic Reticulum Stress Mediates Amyloid β Neurotoxicity via Mitochondrial Cholesterol Trafficking. Am. J. Pathol..

[164] Murley A., Nunnari J. (2016). The Emerging Network of Mitochondria-Organelle Contacts. Mol. Cell.

[165] Vrijsen S., Vrancx C., Del Vecchio M., Swinnen J.V., Agostinis P., Winderickx J., Vangheluwe P., Annaert W. (2022). Inter-Organellar Communication in Parkinson’s and Alzheimer’s Disease: Looking Beyond Endoplasmic Reticulum-Mitochondria Contact Sites. Front. Neurosci..

